# Hypertrophic Cardiomyopathy and Primary Restrictive Cardiomyopathy: Similarities, Differences and Phenocopies

**DOI:** 10.3390/jcm10091954

**Published:** 2021-05-01

**Authors:** Riccardo Vio, Annalisa Angelini, Cristina Basso, Alberto Cipriani, Alessandro Zorzi, Paola Melacini, Gaetano Thiene, Alessandra Rampazzo, Domenico Corrado, Chiara Calore

**Affiliations:** 1 Department of Cardiac, Thoracic and Vascular Sciences and Public Health, University of Padova, 35128 Padova, Italy; riccardo.vio.1@gmail.com (R.V.); annalisa.angelini@unipd.it (A.A.); cristina.basso@unipd.it (C.B.); alberto.cipriani@unipd.it (A.C.); alessandro.zorzi@unipd.it (A.Z.); paola.melacini@unipd.it (P.M.); gaetano.thiene@unipd.it (G.T.); chiara.calore@unipd.it (C.C.); 2 Department of Biology, University of Padova, 35131 Padova, Italy; alessandra.rampazzo@unipd.it; 3 CRIBI Biotechnology Centre, University of Padova, 35131 Padova, Italy

**Keywords:** hypertrophic cardiomyopathy, restrictive cardiomyopathy, cardiomyopathies, restrictive physiology, genetics, heart failure, amyloidosis, Fabry disease, glycogen storage diseases

## Abstract

Hypertrophic cardiomyopathy (HCM) and primary restrictive cardiomyopathy (RCM) have a similar genetic background as they are both caused mainly by variants in sarcomeric genes. These “sarcomeric cardiomyopathies” also share diastolic dysfunction as the prevalent pathophysiological mechanism. Starting from the observation that patients with HCM and primary RCM may coexist in the same family, a characteristic pathophysiological profile of HCM with restrictive physiology has been recently described and supports the hypothesis that familiar forms of primary RCM may represent a part of the phenotypic spectrum of HCM rather than a different genetic cardiomyopathy. To further complicate this scenario some infiltrative (amyloidosis) and storage diseases (Fabry disease and glycogen storage diseases) may show either a hypertrophic or restrictive phenotype according to left ventricular wall thickness and filling pattern. Establishing a correct etiological diagnosis among HCM, primary RCM, and hypertrophic or restrictive phenocopies is of paramount importance for cascade family screening and therapy.

## 1. Introduction

Cardiomyopathies are a heterogeneous group of diseases of the myocardium associated with mechanical and/or electrical dysfunction, due to a variety of causes that are frequently genetic [1]. They can be either confined to the heart (“primary cardiomyopathy”) or part of generalized systemic disorders (“secondary cardiomyopathy”), often leading to cardiovascular death or progressive heart failure-related disability [1]. The most common cardiomyopathy is hypertrophic cardiomyopathy (HCM) caused by variants in sarcomeric (or sarcomere-related) genes, characterized by left ventricular hypertrophy in the absence of another cardiac, systemic, or metabolic disease capable of producing the magnitude of hypertrophy evident [2]. A key pathophysiological aspect of HCM is diastolic dysfunction, which can be severe enough to determine a restrictive left ventricular filling pattern [3], raising problems of differential diagnosis with primary restrictive cardiomyopathy (RCM). Some familial forms of primary RCM have been described in patients carrying variants in sarcomeric genes traditionally associated with HCM [4], and HCM and primary RCM may coexist in the same family [5]. This scenario is further complicated by classifications that group cardiomyopathies based only on ventricular morphology and function irrespective of etiology [6,7]. According to these phenotypic classifications, some infiltrative and storage diseases may be listed as either HCM or RCM depending on the degree of hypertrophy observed and left ventricular filling pattern (hypertrophic or restrictive phenocopies) [7]. This review focuses on morphological, functional, and genetic features of HCM and primary RCM, offering guiding principles for differential diagnosis between them and among cardiomyopathies that can present either with a hypertrophic or restrictive phenotype.

## 2. Hypertrophic Cardiomyopathy

Hypertrophic cardiomyopathy (HCM) is an autosomal dominant heart disease caused by a broad variety of genetic variants involving mostly proteins of the cardiac sarcomere. Given its prevalence of 1:500 in the general population, it is considered the most common genetic cardiovascular disease [1,2]. A left ventricular wall thickness of at least 15 mm (or 13 mm in relatives) is considered sufficient for diagnosis, in the absence of other systemic or cardiac diseases capable of producing the magnitude of hypertrophy evident (e.g., hypertension and aortic stenosis) [2]. At least 1400 variants in numerous genes encoding proteins of the cardiac sarcomere responsible for (or associated with) HCM have been identified [8]. The most commonly involved genes are *MYH7*, *MYBPC3* (these two genes include approximately 80% of all identified variants), *TNNT2*, and *TNNI3*. The majority of patients reveal increased wall thickness by early adulthood [9,10], however different variants can demonstrate substantial variability in age-related penetrance, resulting in delayed expression after the third decade of life, or even beyond mid-life [11,12]. The phenotype in the first-degree family members also shows a wide range of expression, underscoring the importance of other factors including modifier genes and epigenetics [13,14].

Twelve-lead ECG has traditionally been an integral part of non-invasive evaluation of patients with HCM [15,16]. The vast majority of patients show an abnormal ECG tracing, with a reported prevalence of ECG abnormalities of >90% [17]. Left ventricular hypertrophy, ST-segment depression, T wave inversion, pathologic Q waves, left atrial enlargement, and left axis deviation are among the striking ECG abnormalities [15]. Atrial fibrillation is reported in 20% of cases, being associated with heart failure progression [18,19,20]. On the contrary, a normal ECG may predict a less severe HCM phenotype and a better cardiovascular outcome [15,21,22]. Echocardiography is the primary non-invasive imaging technique for diagnosis and risk stratification of HCM [22]. One third of patients have resting left ventricular outflow tract obstruction (>30 mmHg) and another third have latent obstruction that can be unmasked during provocative maneuvers [23]. Mitral regurgitation is a common finding as the result of systolic anterior motion of the mitral valve, and together with diastolic dysfunction leads to the enlargement of the left atrium. Diastolic dysfunction can be severe enough to produce a left ventricular restrictive filling pattern, defined as the ratio of mitral peak velocity of early filling (E) to mitral peak velocity of late filling (A) ≥2 or an E-wave deceleration time ≤ 150 ms. Biagini et al., reported a restrictive left ventricular filling pattern in 5.9% of 239 consecutive HCM patients at baseline, with another 9.1% of them who developed this pattern during follow-up [3]. Another sign of severe diastolic impairment is the triphasic left ventricular filling pattern (showing an additional mid-diastolic filling wave) that can be found in up to one quarter of patients, particularly those with thin-filament gene variants [24]. Systolic function is usually normal or increased, until the development of the end-stage phase. Cardiovascular magnetic resonance (CMR) allows precisely measuring wall thickness (especially at the apex and lateral free wall), cardiac chamber volumes, and left ventricular mass. Myocardial fibrosis is revealed by the injection of gadolinium-based contrast agents in 65% of patients [25,26]. Diffuse and extensive late gadolinium enhancement at CMR, either quantified or estimated by visual inspection (comprising ≥ 15% of left ventricular mass) is now considered an established risk factor for sudden death [2]. Endomyocardial biopsy is not part of the routine diagnostic workup of HCM, but may instead be helpful when myocardial infiltration or storage disease is suspected. Histopathological features of HCM include myocyte disarray, increased interstitial fibrosis and abnormal intramural arterioles with thickened walls and narrowed lumen [27,28,29]. In a large autopsy-based study, coronary artery bridges were a frequent morphological component of phenotypically expressed HCM [30]. 

**Figure 1:**
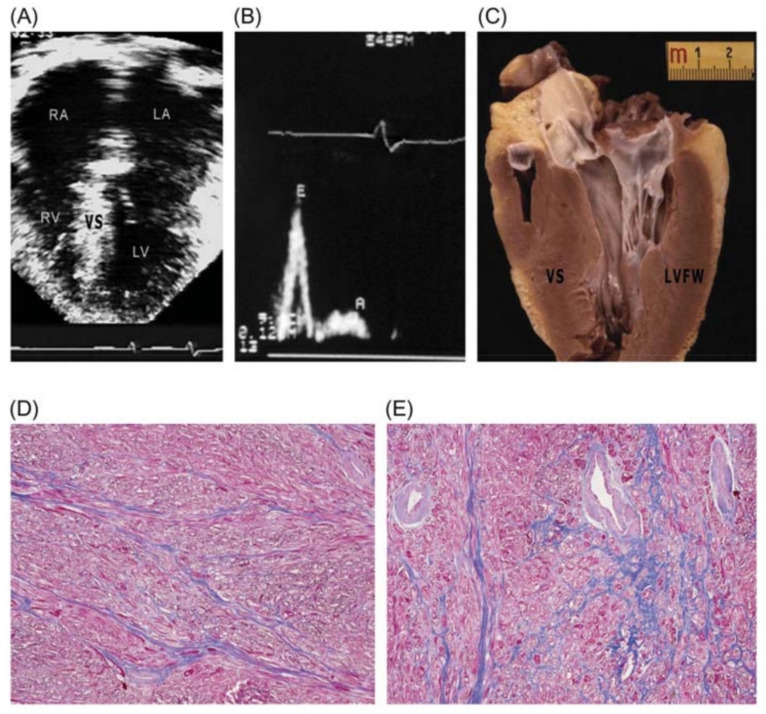
Restrictive form of heart failure due to diastolic dysfunction in a 28-year-old patient with non-obstructive hypertrophic cardiomyopathy preserved systolic function and troponin I variant. (**A**) Four-chamber view in end-diastole showing dilatation of both atria (left atrium, LA = 53 mm), normal-sized ventricles, and mild ventricular septal (VS) thickening (17 mm). (**B**) Pulsed Doppler waveform with evidence of restrictive filling: E/A>2; deceleration time, 150 ms. (**C**) Long-axis left ventricular (LV) plane with mild VS hypertrophy (17 mm); atria missing due to transplantation. (**D**,**E**) LV free wall (**D**) and septum (**E**) showing diffuse myocardial disarray, mild interstitial fibrosis, and intramural small vessel disease. Trichrome stain × 40. LVFW: left ventricular free wall; RA: right atrium; RV: right ventricle. Reproduced with permission from [29].

Patients’ clinical courses are heterogeneous, ranging from benign to sudden cardiac death, being the leading cause of athletic field cardiac arrest in the USA, where it accounts for more than a third of fatalities [31,32]. Different profiles of heart failure in HCM may occur at any age due to diverse pathophysiological mechanisms, including left ventricular outflow obstruction and diastolic or global systolic ventricular dysfunction [23,33,34]. According to Melacini et al., nearly half of patients with advanced heart failure occur in the clinical setting of non-obstructive disease with preserved systolic function, presenting a particularly malignant prognosis with earlier symptom onset [29]. This “restrictive subgroup” of HCM patients show small ventricular cavities, markedly enlarged atria and mild left ventricular hypertrophy with a restrictive left ventricular filling pattern (Figure 1).

**Figure 2:**
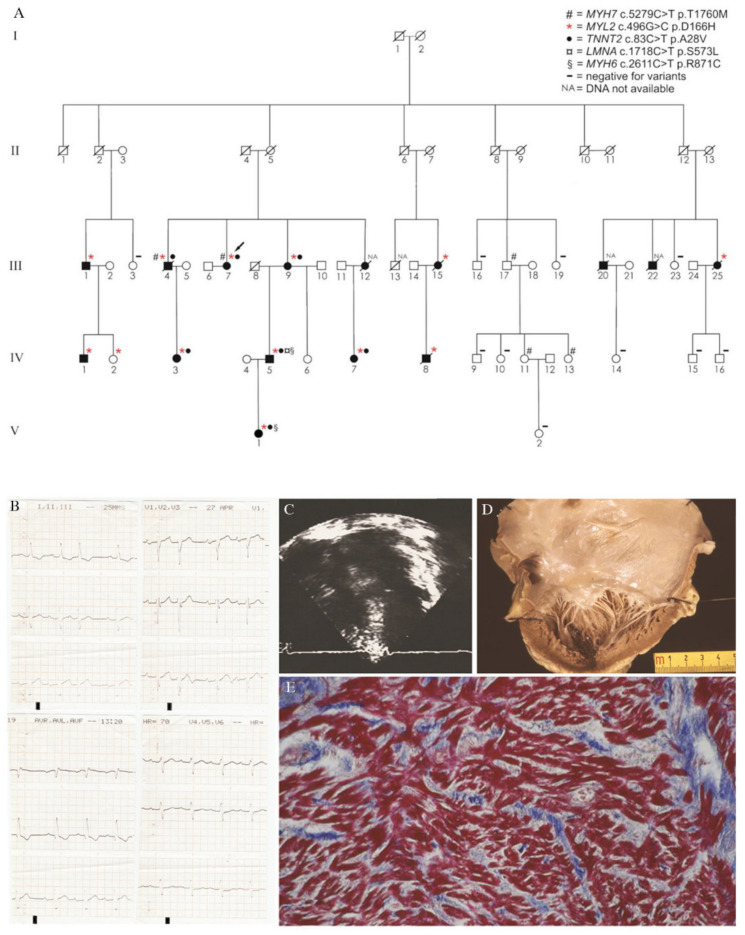
Novel Missense variant in *MYL2* gene and hypertrophic cardiomyopathy associated with a high incidence of restrictive physiology. (**A**) Pedigree of the family. (**B**) Electrocardiogram of subject III-25 showing first-degree AV block and left anterior hemiblock (prior to pacemaker implantation). (**C**) Four-chamber echocardiographic view of subject III-25 showing biatrial enlargement and septal hypertrophy (prior to pacemaker implantation). (**D**) Gross view of the left cardiac chambers of subject III-25: note the severe dilatation of the left atrium with an almost preserved left ventricular volume. The thickness of the LV free wall and ventricular septum are 13 and 14 mm, respectively, in keeping with symmetric mild hypertrophy. (**E**) Histology of the LV free wall of subject III-25: note the diffuse disarray of the cardiac myocytes with tiny interstitial fibrosis (trichrome stain). Modified and reproduced with permission from [36].

When hypertrophy is mild, differential diagnosis with RCM can be difficult and a phenotypic overlap between these two entities may exist. Evaluating 1226 patients from 688 families with HCM, Kubo et al. advanced the “restrictive phenotype” (i.e., no or minimal left ventricular hypertrophy of ≤15 mm and severe diastolic dysfunction) as part of the clinical spectrum of HCM [35]. The authors concluded that the “restrictive phenotype” is an uncommon presentation of HCM (19/1266, 1.5% of cases), associated with severe functional limitation and poor prognosis, resembling idiopathic RCM. Sarcomeric gene variants were identified in eight probands, including variants in *MYH7* and *TNNI3*. However, not all family members with the same variant developed the “restrictive phenotype” suggesting that other genetic (e.g., modifier genes), epigenetic or environmental factors are involved. Our research group recently reported a novel missense variant in the *MYL2* gene associated with HCM showing high incidence of restrictive physiology (Figure 2) [36]. Specifically, 38% of *MYL2* carriers in our kindred had a restrictive filling pattern, much higher than previously reported [3], and one patient showed the “restrictive phenotype” defined by Kubo et al. [35]. Probably linked to the severe diastolic dysfunction, up to 62% of *MYL2* carriers experienced atrial fibrillation and many patients had a poor clinical outcome.

## 3. Primary Restrictive Cardiomyopathy

Primary RCM is a rare myocardial disease characterized by normal or decreased volume of both ventricles associated with biatrial enlargement, normal left ventricular wall thickness, impaired ventricular filling with restrictive physiology, and normal or near-normal systolic function [1]. Since the first report of a familial form caused by a cardiac troponin I *(TNNI3)* variant [4], primary RCM was believed to be a sarcomeric disease and was labeled together with HCM as a sarcomeric cardiomyopathy (“sarcomyopathies”) according to a genomic/postgenomic classification [37,38,39,40]. Successively, apart from a small subset of rare nonsarcomeric variants (myopalladin, filament-C, desmin, and alpha-beta crystallin [41,42,43,44]), mounting evidence supported this view. Moreover, “desminopathies” (i.e., familial RCM with variants in the desmin gene) should be classified as a separate and secondary cardiomyopathy, given the almost invariable skeletal muscle involvement producing a skeletal myopathy [43]. The most common sarcomeric gene variants associated with primary RCM involve troponin T (*TNNT2*), alpha cardiac actin (*ACTC*), myosin-binding protein C (*MYBPC3*), tropomyosin 1 (*TPM1*), and myosin light chain 2 (*MYL2*) and 3 (*MYL3*) [45,46,47,48]. Moreover, *TNNI3*, *MYH7,* and *MYL2* variants were reported as responsible for some familial cases of HCM with “restrictive phenotype” (phenotypes diagnostic of RCM) [4,36]. The coexistence of HCM and RCM phenotypes in the same families with the same disease-causing variants led to the hypothesis that familiar forms of RCM may represent a part of the phenotypic spectrum of HCM rather than a different genetic cardiomyopathy [5,49]. 

The phenotypic expression of other cardiomyopathies (e.g., infiltrative and storage disorders, endomyocardial fibrosis and sarcoidosis) may mimic primary RCM [1,50,51]. However, in primary RCM the morphologic and hemodynamic abnormalities occur in the absence of specific histopathological changes [50,51] and possible findings include interstitial fibrosis, myocyte hypertrophy, and myocardial disarray as in HCM [5,38,52]. 

**Figure 3:**
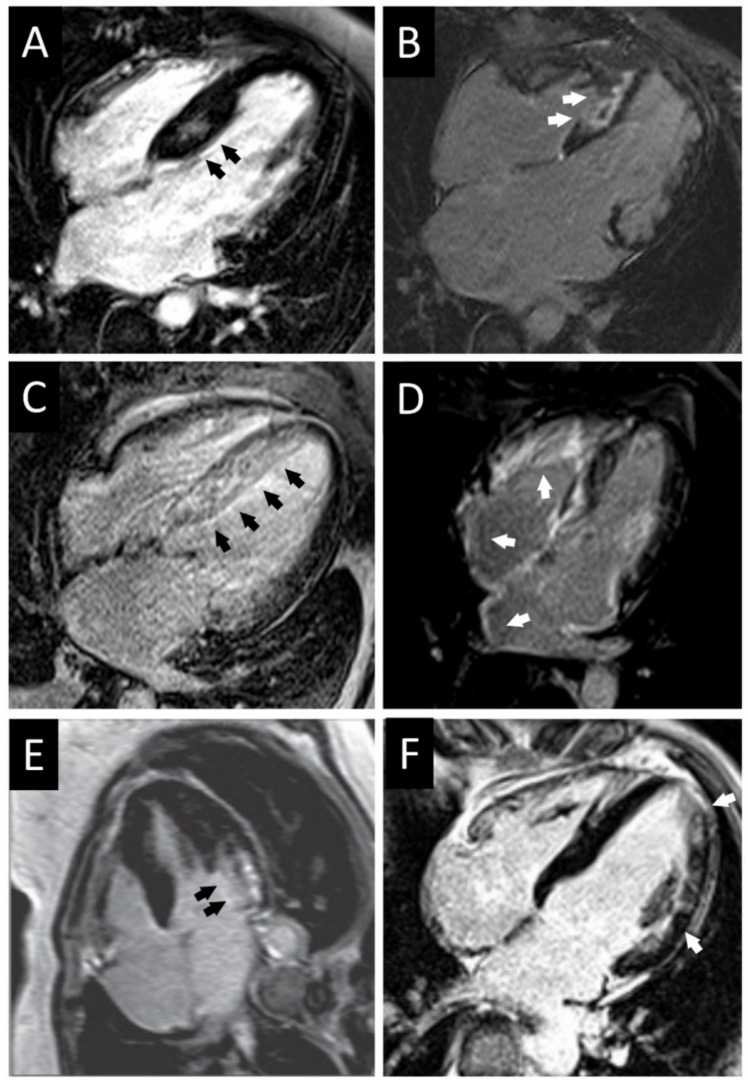
Representative cardiac magnetic resonance findings of patients with obstructive hypertrophic cardiomyopathy, hypertrophic cardiomyopathy with restrictive phenotype, amyloidosis, and Fabry disease. (**A**) Obstructive HCM with intramural septal LGE. (**B**) HCM with restrictive physiology (*MYH7* variant) with massive septal fibrosis and severe atrial enlargement. (**C**) Light chain immunoglobulin (AL) amyloidosis with transmural septal LGE. (**D**) Wild-type transthyretin (ATTRwt) amyloidosis with LGE particularly in the right ventricle and atria. (**E**) Fabry disease with hypertrophic phenotype and subendocardial LGE at the basal lateral segment of the left ventricle. (**F**) Fabry disease with mild hypertrophy and intramural LGE at the mid-lateral segment of the left ventricle and apex. HCM: hypertrophic cardiomyopathy; LGE: late gadolinium enhancement.

The key aspect of primary RCM is the impairment of the ventricular filling dynamics that ultimately lead to the increase of ventricular end-diastolic pressures and atrial dilation. Such diastolic impairment is responsible for the development of progressive heart failure symptoms (dyspnea, fatigue, and exercise intolerance). Cardiac catheterization shows increased ventricular filling pressures with the typical dip-and-plateau or square-root sign; left ventricular diastolic pressure is usually 5 mmHg higher compared to the right one due to imbalanced involvement and compliance of the two chambers [51]. Equalization of ventricular diastolic pressures is more typical of constrictive pericarditis but it may be present and does not rule out the diagnosis of primary RCM [51,53]. Differential diagnosis between these two conditions is usually straightforward: history of chronic pericarditis, cardiac surgery, or chest irradiation favors the diagnosis of a constrictive pericarditis. Non-invasive diagnostic testing (echocardiography, CMR, and cardiac computed tomography (CT)) or invasive hemodynamic study are able to unveil characteristic features of constrictive pericarditis including a thickened pericardium (>4 mm), interventricular dependence, and dissociation between intracardiac and intrathoracic pressures during respiration [53]. CMR is also helpful to differentiate primary RCM with secondary forms of RCM due to infiltrative or storage diseases which show typical radiological findings (see below) (Figure 3). Fluorodeoxyglucose (FDG) positron emission tomography (PET)/CT is indicated in case of clinical suspicion of sarcoidosis [54]. 

As happened for all the other principal cardiomyopathies, before the understanding of molecular genetics of the disease primary RCM was referred to as “idiopathic” RCM [50]. The largest series is derived from the study of Ammash et al., who described 94 cases of idiopathic RCM collected between 1979 and 1996, after excluding patients with known ischemic, hypertensive, valvular, congenital, or pericardial heart disease and other conditions such as amyloidosis, hemochromatosis, and eosinophilic syndrome [55]. According to the study results, idiopathic RCM can occur at any age and mostly in women. The ECG was almost invariably abnormal, with the majority of patients showing atrial fibrillation (74%) and nonspecific ST-T wave abnormalities (80%); intraventricular conduction delay was reported in 19% of patients and premature ventricular beats in 14%. Of note, none of the patients had left ventricular hypertrophy (typical of HCM) or low QRS voltages (typical of amyloidosis, see below). At the echocardiogram all patients showed biatrial enlargement, nondilated ventricles with normal wall thickness; transmitral valve Doppler showed either E-wave deceleration time of <150 ms or an E/A ratio > 2. None of the biopsy specimens demonstrated amyloid or iron deposition, caseating granulomas, eosinophilic or lymphocytic infiltrates, or any interstitial inflammatory changes.

## 4. Cardiomyopathies with either Hypertrophic or Restrictive Phenotype

**Figure 4:**
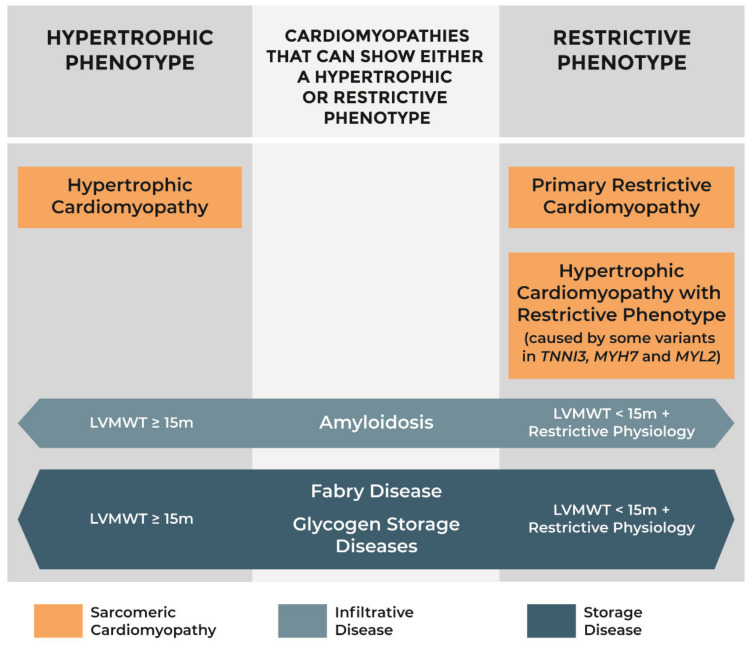
Phenotypes of hypertrophic cardiomyopathy, primary restrictive cardiomyopathy, amyloidosis, Fabry disease, and glycogen storage diseases. LVMWT: left ventricular maximal wall thickness.

Some infiltrative and storage diseases may present as phenocopies of either HCM or primary RCM, according to the degree of hypertrophy observed and left ventricular filling pattern (Figure 4). If maximal left ventricular wall thickness is at least 15 mm, they must be differentiated from sarcomeric HCM. Otherwise, the restrictive physiology inherent to the pathological process of myocardial infiltration or storage yields a phenotype similar to that of primary RCM. Unmasking the underlying disease and going beyond the ventricular morphology and function, is crucial in order to establish prognosis, guide reproductive choices, and offer specific therapy to the patient. Age of onset is among the factors to consider for differential diagnosis: for instance, glycogen storage diseases are more common in infants, whereas ATTRwt amyloidosis involves predominantly men over the age of 65 years [56,57]. 

Herein we describe the cardiomyopathies that can show either a hypertrophic or a restrictive phenotype. Table 1 reports the full list of HCM and primary RCM phenocopies, and Table 2 summarizes the common diagnostic findings of HCM, primary RCM, amyloidosis, Fabry disease, and glycogen storage diseases.

### 4.1. Amyloidosis

Amyloidosis is a systemic syndrome that represents the archetype of the infiltrative form of RCM. Extracellular deposition of insoluble amyloid fibrils involves a variety of organs including the heart, leading to cardiomyocyte separation, cellular toxicity, and apoptosis. Overall, these morphological changes increase myocardial stiffness causing diastolic disfunction and heart failure. The three most common types of amyloidosis affecting the heart (“cardiac amyloidosis”) are AL, ATTRwt, and mutant transthyretin (ATTRm) amyloidosis. AL amyloidosis is a rare hematological disorder, in which there is an increased production of monoclonal kappa or lambda light chains that tend to misfold favoring tissue deposition. Transthyretin is a protein with tetrameric configuration, produced mainly in the liver; its monomers have amyloidogenic properties. ATTRm amyloidosis is a heritable autosomal disorder caused by variants in the *TTR* gene that increase the likelihood of the TTR tetramer to dissociate into monomers and form amyloid fibrils. In the absence of *TTR* pathogenic variants, spontaneous dissociation of TTR tetramers may occur (usually later in life), determining ATTRwt amyloidosis (senile amyloidosis). AL amyloidosis is rare and has an estimated prevalence of 8 to 12 per million [59,60,61], although emerging data suggest that ATTRwt amyloidosis is not uncommon: it may affect up to 13% of patients hospitalized for heart failure with preserved ejection fraction [62]. Variants in the *TTR* gene causing ATTRm amyloidosis are relatively rare but are endemic in some geographic regions (e.g., Portugal and Sweden) [63].

Clinical presentation is useful, per se, in the differential diagnosis with sarcomeric cardiomyopathies, as patients with amyloidosis show variable extracardiac organ involvement. Peripheral and autonomic neuropathy, carpal tunnel syndrome, macroglossia, nephropathy, and hepatopathy are red flags for amyloidosis and should be sought in all patients [64]. At the ECG, low voltages are reported in 20–60% of patients and mostly in those with AL amyloidosis [65,66]. Pseudoinfarction patterns (Q waves or QS complexes) are common (70% of cases), as they also are in HCM [15,67]. A significant percentage of patients with amyloidosis develop progressive atrioventricular conduction disturbances or bundle branch blocks [67]. Echocardiography reveals a concentric thickening of the left ventricular free wall and septum, that is usually greater in patients with ATTRwt amyloidosis [68]. Unlike HCM, right ventricular free wall, valves and atrial septum are also thickened. According to a study by Quarta et al. who analyzed 172 patients with AL, ATTRm or ATTRwt amyloidosis, a third of the overall population (*n* = 63) showed a mean left ventricular wall thickness (almost identical to the interventricular septum thickness given the concentric hypertrophy) of ≤15 mm (range 11.5–15 mm), and the other two thirds >15 mm [68]. A restrictive filling pattern was found in 35% of patients. From these data it is clear how the phenotype of patients with amyloidosis may mimic either HCM or primary RCM. Some peculiar although nonspecific echocardiographic findings of amyloidosis that may guide differential diagnosis are the following: right ventricular free wall, valves and atrial septum thickening, pericardial effusion and “granular” appearance of the myocardium [69]. Early signs of cardiac amyloid infiltration are the reduced basal longitudinal strain compared to the normal or supranormal apical longitudinal strain (“apical sparing”) and low septal and lateral early diastolic mitral annular velocities (E’-wave) at tissue-Doppler echocardiography [64,70]. CMR can provide further clues to the diagnosis of amyloidosis by showing a characteristic diffuse subendocardial late gadolinium enhancement (“zebra pattern”), and difficulty in nulling the myocardial signal on phase sensitive inversion recovery sequences [71,72,73]. Bone scintigraphy imaging has recently gained crucial relevance, since it has high specificity and a positive predictive value of 100% for transthyretin amyloidosis in case of moderate to high tracer uptake [64]. In the absence of detectable monoclonal protein in serum or urine, bone scintigraphy allows non-histological diagnosis of transthyretin amyloidosis [74]. However, tissue diagnosis remains the gold standard. Congo red staining binds to the deposits of amyloid fibrils and yields characteristic apple-green birefringence under polarized light microscopy. Electron microscopy demonstrates randomly oriented and non-branching fibrils and subsequently immunohistochemistry or laser microdissection/mass spectroscopy allows the subtyping of amyloid fibril [75,76].

The treatment of amyloidosis goes beyond the purpose of the present review, but novel specific therapies improving cardiac and neurological outcomes have been recently approved and its recognition is therefore of paramount importance [77,78]. 

### 4.2. Storage Diseases

#### 4.2.1. Fabry Disease 

Fabry disease is a lysosomal storage disease with an X-linked recessive inheritance caused by variants in the *GLA* gene, that determine an absent or deficient activity of lysosomal alpha galactosidase A. As a result, globotriaosylceramide accumulate within virtually all cell types’ lysosomes, leading to cellular death, inflammation, oxidative stress, and fibrosis; cardiomyocyte hypertrophy results from increased concentrations of sphingolipid and vascular smooth muscle cell proliferation [79]. The prevalence of Fabry disease is estimated to be 1/3000–1/8000 newborns [80,81]. Indeed, near 1% of HCM population can be newly diagnosed with Fabry disease when screened for this condition [82]. First signs of the disease appear during childhood or early adulthood in men; women are diagnosed later in life and show a better prognosis, considering the pattern of inheritance of the disease (X-linked recessive transmission) [79]. In adults, clinical signs of Fabry disease include a variable combination of kidney, neurological, and cardiac dysfunctions. Cardiac symptoms occur in the majority of patients, and include dyspnea, heart failure, angina caused by microvascular dysfunction, palpitations, and syncope [83]. Patients with Fabry disease most often present with left ventricular hypertrophy, sometimes as a predominant or isolated feature (“cardiac variant”) [81,84]. Left ventricular hypertrophy is the most frequent cardiac sign, reported in over a half of men and one third of women [83]. Other diagnostic red flags for Fabry disease are kidney disease, stroke, angiokeratoma corporis diffusum, and cornea verticillate [79,85]. Arrhythmic manifestations include chronotropic incompetence, sinus node dysfunction, advanced atrioventricular block, atrial fibrillation, ventricular tachycardia, and ventricular fibrillation leading to sudden death [86].

The electrocardiogram often displays increased QRS voltages as the result of the deposition within the cardiomyocytes rather than in the interstitium as it occurs in infiltrative disease (e.g., amyloidosis) characterized on the contrary by low QRS voltages. Other ECG changes that can be seen in Fabry disease patients include a short PR interval, sinus bradycardia, atrioventricular conduction disturbances, bundle branch blocks, pathologic Q waves, negative T waves, and atrial fibrillation [81,83,87,88]. Supraventricular and ventricular arrhythmias are common in Fabry disease and may occur even in the prehypertrophic phase before any clinical or cardiac imaging abnormalities [81,89]. At the echocardiogram, left ventricular hypertrophy is frequently encountered (almost invariably concentric), without left ventricular outflow obstruction or systolic dysfunction [90]. However, asymmetrical hypertrophy and subaortic obstruction have also been reported, mimicking the phenotypical and clinical features of sarcomeric HCM [91]. Other characteristics that may be documented are left atrial enlargement, valvular thickening and right ventricular hypertrophy [90,91]. These latter findings are similar to those of patients with amyloidosis, but further similarities between Fabry disease and amyloidosis exist. In fact, even in Fabry disease the reduced tissue-Doppler velocities at the mitral annulus can be the earliest signs of intrinsic myocardial relaxation impairment, preceding left ventricular hypertrophy [92]. Diastolic dysfunction progressively worsens until the development of a restrictive filling pattern [79]. Mid-mural late gadolinium enhancement on basal segment of the inferolateral left ventricular free wall is a typical finding at the CMR, often occurring on a non-hypertrophied wall [93]. Conversely, in HCM the site of predilection for LGE accumulation is the part with the greater hypertrophy [94]. Moreover, T1 mapping helps in the refinement of differential diagnosis with either HCM or amyloidosis. Sphingolipid storage in the myocardium yields low native T1 values which are specific for Fabry disease, as opposed to the other two conditions [95,96]. Definite diagnosis of Fabry disease is reached through the demonstration of decreased/absent serum or leukocyte alpha galactosidase A activity or pathogenic variants in the *GLA* gene. In addition, endomyocardial biopsy can identify histological alterations by showing the accumulation of the degradation product of globotriaosylceramide in the sarcoplasm of myocytes, forming concentric lamellar bodies [85,97]. Although the clinical response is not always optimal, enzyme replacement therapy may ameliorate symptoms and renal function, potentially reducing left ventricular hypertrophy, cardiac events, stroke, and overall death [98,99,100,101].

#### 4.2.2. Glycogen Storage Diseases

Glycogen storage diseases are a group of inherited genetic disorders that cause glycogen to be improperly accumulated in the body. Although they are listed as cardiomyopathies that can also have a restrictive phenotype [7,51], patients affected with glycogen storage diseases typically show a marked left ventricular hypertrophy and must be differentiated mainly from HCM. This is particularly true for Danon disease (caused by deficiency of lysosome-associated membrane protein 2-LAMP 2) in which the hypertrophy can be so extreme as to reach 65 mm of left ventricular maximal wall thickness [102]. These morphological findings correspond to increased QRS voltages and T wave abnormalities at the ECG. Excess glycogen accumulates in cardiomyocytes and skeletal muscle fibers leading to formation of vacuoles that stain positive with periodic Acid Schiff [85]. In addition to skeletal and cardiac muscle involvement, mild to moderate mental retardation is frequently observed.

PRKAG2 syndrome is another type of glycogen storage disease, whose presentation includes skeletal myopathy, marked cardiac hypertrophy, arrhythmias, and conduction defects [85]. Left ventricular hypertrophy is often progressive and associated with both diastolic and systolic heart failure [103]. Maximal ventricular wall thickness varies widely among different patients, ranging from normal values to over 40 mm [104,105,106]. A restrictive filling pattern, left ventricular outflow tract obstruction, and dilated progression are leading causes of cardiac transplant or death [104,105,107]. Its key aspect is the association with Wolff–Parkinson–White syndrome (characterized by a short PR interval, ventricular pre-excitation, and supraventricular tachycardia), together with possible advanced atrioventricular blocks requiring pacemaker implantation. CMR patients with PRKAG2 syndrome may show LGE only in the advanced stages, together with high T1 values caused by fibrosis [108].

The heart is also part of the clinical spectrum of Pompe disease (acid maltase deficiency or glycogen storage disease type II) [57]. This disorder usually has an infantile onset, producing massive cardiac hypertrophy in neonatal and pediatric ages, able to determine left ventricular outflow tract obstruction. Diastolic dysfunction is generally present and systolic dysfunction can be seen later on [57]. Adult-onset acid maltase deficiency is a rare variant of the disease: as it seldom affects the heart, the cardiac phenotype of adults with this condition is poorly characterized [109]. The morphology of the heart of patients diagnosed with glycogen storage disease type III, also known as Cori disease, usually mimics HCM but can show restrictive physiology [51,110].

## 5. Conclusions

HCM and primary RCM have a similar genetic background as they are both sarcomeric cardiomyopathies, and significant phenotypic overlap can exist between them. Patients with HCM and primary RCM may coexist in the same family and a new category of HCM with restrictive physiology has been recently described. One hypothesis is that familiar forms of primary RCM may represent a part of the phenotypic spectrum of HCM rather than a different genetic cardiomyopathy. To further complicate this delicate scenario some infiltrative and storage diseases may show either a hypertrophic or restrictive phenotype (HCM and primary RCM phenocopies) according to left ventricular wall thickness and filling pattern. Establishing a correct diagnosis is of paramount importance for cascade family screening and therapy.

## Figures and Tables

**Table 1:** Phenocopies of hypertrophic cardiomyopathy and primary restrictive cardiomyopathy.

Phenocopies	
Hypertrophic Cardiomyopathy	Primary Restrictive Cardiomyopathy
Infiltrative diseases: **-Amyloidosis**	Infiltrative diseases: **-Amyloidosis**
Storage diseases: **-Fabry disease** **-Glycogen storage diseases**	Storage diseases: **-Fabry disease** **-Glycogen storage diseases** -Haemochromatosis
RASopathies *: -Noonan syndrome -LEOPARD syndrome -Costello syndrome -Cardiofaciocutaneous syndrome	Endomyocardial diseases: -Endomyocardial fibrosis -Hypereosinophilic syndrome
Mitochondrial diseases (MELAS)	Sarcoidosis
Carnitine disorders	Scleroderma
Friederich’s ataxia	Pseudoxanthoma elasticum
Beckwith–Wiedemann syndrome	Carcinoid heart disease
Infant of diabetic mother	Metastatic cancers
Drugs (tacrolimus, hydroxychloroquine, steroids)	Drugs (anthracyclines)
	Radiation

Cardiomyopathies highlighted in bold can show either a hypertrophic or restrictive phenotype. * Developmental disorders caused by germline variants in genes that encode components or regulators of the Ras/MAPK pathway [58].

**Table 2:** Common diagnostic findings of hypertrophic cardiomyopathy, primary restrictive cardiomyopathy, amyloidosis, Fabry disease, and glycogen storage diseases.

	HCM	Primary RCM	Amyloidosis	Fabry Disease	Glycogen Storage Diseases
ECG	Increased QRS voltages, ST-T wave changes, pathologic Q waves, LAE, LAD	Normal QRS voltages, ST-T wave changes, atrial fibrillation, intraventricular conduction delay	Low QRS voltages, Q waves and QS complexes, AV blocks and bundle branch blocks	Increased QRS voltages, short PR interval, pathologic Q waves, T wave inversion, sinus bradycardia, AV blocks, bundle branch blocks	Increased QRS voltages, short PR interval, T wave abnormalities, AV blocks
Echo	Mild to severe asymmetrical, concentric or apical hypertrophy. LVOT obstruction. Left atrial enlargement. Mitral regurgitation. Diastolic dysfunction (from mild to restrictive physiology)	Nondilated ventricles with normal wall thickness, biatrial enlargement, restrictive filling pattern	Mild concentric left ventricular hypertrophy, right ventricular hypertrophy, thickening of valves and atrial septum, pericardial effusion, “granular” appearance of myocardium, “apical sparing” at global longitudinal strain, restrictive filling pattern	Concentric left ventricular hypertrophy without LVOT obstruction. Left atrial enlargement, valvular thickening, right ventricular hypertrophy. Diastolic dysfunction (from mild to restrictive physiology)	Normal to extreme left ventricular hypertrophy with possible LVOT obstruction, diastolic dysfunction, and restrictive filling pattern
CMR	LGE in most hypertrophied regionsHigh native T1 values	-	Diffuse subendocardial LGE (“zebra pattern”), difficulty in nulling the myocardial signal on phase sensitive inversion recovery sequences. High native T1 values	Mid-mural LGE on basal segment of non-hypertrophied inferolateral wall. Low native T1 values	LGE and high T1 values in the advanced stage of the disease
EMB	Myocyte hypertrophy, myocardial disarray, interstitial fibrosis	Interstitial fibrosis, myocyte hypertrophy, myocardial disarray	Apple-green birefringence under polarized light microscopy using Congo red staining. Randomly oriented and non-branching fibrils at electron microscopy	Concentric lamellar bodies (degraded products of globotriaosylceramide in the sarcoplasm)	Vacuoles containing glycogen that stain positive with periodic Acid Schiff

ECG: electrocardiogram; Echo: echocardiogram; CMR: cardiac magnetic resonance; EMB: endomyocardial biopsy; LAE: left atrial enlargement; LAD: left axis deviation; LVOT: left ventricular outflow tract; LGE: late gadolinium enhancement; AV: atrioventricular.

## Data Availability

No new data were created or analyzed in this study. Data sharing is not applicable to this article.

## References

[1] Maron B.J., Towbin J.A., Thiene G., Antzelevitch C., Corrado D., Arnett D., Moss A.J., Seidman C.E., Young J.B., American Heart Association (2006). Contemporary definitions and classification of the cardiomyopathies: An American Heart Association Scientific Statement from the Council on Clinical Cardiology, Heart Failure and Transplantation Committee; Quality of Care and Outcomes Research and Functional Genomics and Translational Biology Interdisciplinary Working Groups; and Council on Epidemiology and Prevention. Circulation.

[2] Ommen S.R., Mital S., Burke M.A., Day S.M., Deswal A., Elliott P., Evanovich L.L., Hung J., Joglar J.A., Kantor P. (2020). 2020 AHA/ACC Guideline for the Diagnosis and Treatment of Patients with Hypertrophic Cardiomyopathy: A Report of the American College of Cardiology/American Heart Association Joint Committee on Clinical Practice Guidelines. Circulation.

[3] Biagini E., Spirito P., Rocchi G., Ferlito M., Rosmini S., Lai F., Lorenzini M., Terzi F., Bacchi-Reggiani L., Boriani G. (2009). Prognostic Implications of the Doppler Restrictive Filling Pattern in Hypertrophic Cardiomyopathy. Am. J. Cardiol..

[4] Mogensen J., Kubo T., Duque M., Uribe W., Shaw A., Murphy R., Gimeno J.R., Elliott P., McKenna W.J. (2003). Idiopathic restrictive cardiomyopathy is part of the clinical expression of cardiac troponin I mutations. J. Clin. Investig..

[5] Angelini A., Calzolari V., Thiene G., Boffa G.M., Valente M., Daliento L., Basso C., Calabrese F., Razzolini R., Livi U. (1997). Morphologic spectrum of primary restrictive cardiomyopathy. Am. J. Cardiol..

[6] Richardson P., McKenna W., Bristow M., Maisch B., Mautner B., O’Connell J., Olsen E., Thiene G., Goodwin J., Gyarfas I. (1996). Report of the 1995 World Health Organization/International Society and Federation of Cardiology Task Force on the definition and classification of cardiomyopathies. Circulation.

[7] Elliott P., Andersson B., Arbustini E., Bilinska Z., Cecchi F., Charron P., Dubourg O., Kühl U., Maisch B., McKenna W.J. (2008). Classification of the cardiomyopathies: A position statement from the european society of cardiology working group on myocardial and pericardial diseases. Eur. Heart J..

[8] Maron B.J., Maron M.S., Semsarian C. (2012). Genetics of hypertrophic cardiomyopathy after 20 years: Clinical perspectives. J. Am. Coll. Cardiol..

[9] Maron B.J., Spirito P., Wesley Y., Arce J. (1986). Development and progression of left ventricular hypertrophy in children with hypertrophic cardiomyopathy. N. Engl. J. Med..

[10] Spirito P., Maron B.J. (1987). Absence of progression of left ventricular hypertrophy in adult patients with hypertrophic cardiomyopathy. J. Am. Coll. Cardiol..

[11] Maron B.J., Niimura H., Casey S.A., Soper M.K., Wright G.B., Seidman J.G., Seidman C.E. (2001). Development of left ventricular hypertrophy in adults with hypertrophic cardiomyopathy caused by cardiac myosin-binding protein C gene mutations. J. Am. Coll. Cardiol..

[12] Calore C., De Bortoli M., Romualdi C., Lorenzon A., Angelini A., Basso C., Thiene G., Iliceto S., Rampazzo A., Melacini P. (2015). A founder MYBPC3 mutation results in HCM with a high risk of sudden death after the fourth decade of life. J. Med. Genet..

[13] Alcalai R., Seidman J.G., Seidman C.E. (2008). Genetic basis of hypertrophic cardiomyopathy: From bench to the clinics. J. Cardiovasc. Electrophysiol..

[14] Marian A.J., Braunwald E. (2017). Hypertrophic Cardiomyopathy: Genetics, Pathogenesis, Clinical Manifestations, Diagnosis, and Therapy. Circ. Res..

[15] Zorzi A., Calore C., Vio R., Pelliccia A., Corrado D. (2018). Accuracy of the ECG for differential diagnosis between hypertrophic cardiomyopathy and athlete’s heart: Comparison between the European Society of Cardiology (2010) and International (2017) criteria. Br. J. Sports Med..

[16] Zorzi A., Vio R., Bettella N., Corrado D. (2020). Criteria for interpretation of the athlete’s ECG: A critical appraisal. Pacing Clin. Electrophysiol..

[17] Calore C., Melacini P., Pelliccia A., Cianfrocca C., Schiavon M., Di Paolo F.M., Bovolato F., Quattrini F.M., Basso C., Thiene G. (2013). Prevalence and clinical meaning of isolated increase of QRS voltages in hypertrophic cardiomyopathy versus athlete’s heart: Relevance to athletic screening. Int. J. Cardiol..

[18] Guttmann O.P., Rahman M.S., O’Mahony C., Anastasakis A., Elliott P.M. (2014). Atrial fibrillation and thromboembolism in patients with hypertrophic cardiomyopathy: Systematic review. Heart.

[19] Maron B.J., Olivotto I., Bellone P., Conte M.R., Cecchi F., Flygenring B.P., Casey S.A., Gohman T.E., Bongioanni S., Spirito P. (2002). Clinical profile of stroke in 900 patients with hypertrophic cardiomyopathy. J. Am. Coll. Cardiol..

[20] Rowin E.J., Hausvater A., Link M.S., Abt P., Gionfriddo W., Wang W., Rastegar H., Estes N.A.M., Maron M.S., Maron B.J. (2017). Clinical Profile and Consequences of Atrial Fibrillation in Hypertrophic Cardiomyopathy. Circulation.

[21] McLeod C.J., Ackerman M.J., Nishimura R.A., Tajik A.J., Gersh B.J., Ommen S.R. (2009). Outcome of Patients with Hypertrophic Cardiomyopathy and a Normal Electrocardiogram. J. Am. Coll. Cardiol..

[22] Spirito P., Maron B.J. (1990). Relation between extent of left ventricular hypertrophy and occurrence of sudden cardiac death in hypertrophic cardiomyopathy. J. Am. Coll. Cardiol..

[23] Maron M.S., Olivotto I., Zenovich A.G., Link M.S., Pandian N.G., Kuvin J.T., Nistri S., Cecchi F., Udelson J.E., Maron B.J. (2006). Hypertrophic cardiomyopathy is predominantly a disease of left ventricular outflow tract obstruction. Circulation.

[24] Coppini R., Ho C.Y., Ashley E., Day S., Ferrantini C., Girolami F., Tomberli B., Bardi S., Torricelli F., Cecchi F. (2014). Clinical phenotype and outcome of hypertrophic cardiomyopathy associated with thin-filament gene mutations. J. Am. Coll. Cardiol..

[25] Rudolph A., Abdel-Aty H., Bohl S., Boyé P., Zagrosek A., Dietz R., Schulz-Menger J. (2009). Noninvasive Detection of Fibrosis Applying Contrast-Enhanced Cardiac Magnetic Resonance in Different Forms of Left Ventricular Hypertrophy. Relation to Remodeling. J. Am. Coll. Cardiol..

[26] Green J.J., Berger J.S., Kramer C.M., Salerno M. (2012). Prognostic value of late gadolinium enhancement in clinical outcomes for hypertrophic cardiomyopathy. JACC Cardiovasc. Imaging.

[27] Maron B.J., Roberts W.C. (1979). Quantitative analysis of cardiac muscle cell disorganization in the ventricular septum of patients with hypertrophic cardiomyopathy. Circulation.

[28] Shirani J., Pick R., Roberts W.C., Maron B.J. (2000). Morphology and significance of the left ventricular collagen network in young patients with hypertrophic cardiomyopathy and sudden cardiac death. J. Am. Coll. Cardiol..

[29] Melacini P., Basso C., Angelini A., Calore C., Bobbo F., Tokajuk B., Bellini N., Smaniotto G., Zucchetto M., Iliceto S. (2010). Clinicopathological profiles of progressive heart failure in hypertrophic cardiomyopathy. Eur. Heart J..

[30] Basso C., Thiene G., Mackey-Bojack S., Frigo A.C., Corrado D., Maron B.J. (2009). Myocardial bridging, a frequent component of the hypertrophic cardiomyopathy phenotype, lacks systematic association with sudden cardiac death. Eur. Heart J..

[31] Corrado D., Basso C., Thiene G. (2005). Essay: Sudden death in young athletes. Lancet.

[32] Corrado D., Migliore F., Basso C., Thiene G. (2006). Exercise and the risk of sudden cardiac death. Herz.

[33] Maron B.J. (2002). Hypertrophic cardiomyopathy: A systematic review. JAMA.

[34] Harris K.M., Spirito P., Maron M.S., Zenovich A.G., Formisano F., Lesser J.R., Mackey-Bojack S., Manning W.J., Udelson J.E., Maron B.J. (2006). Prevalence, clinical profile, and significance of left ventricular remodeling in the end-stage phase of hypertrophic cardiomyopathy. Circulation.

[35] Kubo T., Gimeno J.R., Bahl A., Steffensen U., Steffensen M., Osman E., Thaman R., Mogensen J., Elliott P.M., Doi Y. (2007). Prevalence, Clinical Significance, and Genetic Basis of Hypertrophic Cardiomyopathy with Restrictive Phenotype. J. Am. Coll. Cardiol..

[36] De Bortoli M., Vio R., Basso C., Calore M., Minervini G., Angelini A., Melacini P., Vitiello L., Vazza G., Thiene G. (2020). Novel Missense Variant in MYL2 Gene Associated with Hypertrophic Cardiomyopathy Showing High Incidence of Restrictive Physiology. Circ. Genomic. Precis. Med..

[37] Thiene G., Corrado D., Basso C. (2004). Cardiomyopathies: Is it time for a molecular classification?. Eur. Heart J..

[38] Thiene G., Basso C., Calabrese F., Angelini A., Valente M. (2005). Twenty years of progress and beckoning frontiers in cardiovascular pathology: Cardiomyopathies. Cardiovasc. Pathol..

[39] Thiene G., Corrado D., Basso C. (2008). Revisiting definition and classification of cardiomyopathies in the era of molecular medicine. Eur. Heart J..

[40] McKenna W.J., Maron B.J., Thiene G. (2017). Classification, epidemiology, and global burden of cardiomyopathies. Circ. Res..

[41] Purevjav E., Arimura T., Augustin S., Huby A.C., Takagi K., Nunoda S., Kearney D.L., Taylor M.D., Terasaki F., Bos J.M. (2012). Molecular basis for clinical heterogeneity in inherited cardiomyopathies due to myopalladin mutations. Hum. Mol. Genet..

[42] Brodehl A., Ferrier R.A., Hamilton S.J., Greenway S.C., Brundler M.A., Yu W., Gibson W.T., McKinnon M.L., McGillivray B., Alvarez N. (2016). Mutations in FLNC are Associated with Familial Restrictive Cardiomyopathy. Hum. Mutat..

[43] Zhang J., Kumar A., Stalker H.J., Virdi G., Ferrans V.J., Horiba K., Fricker F.J., Wallace M.R. (2001). Clinical and molecular studies of a large family with desmin-associated restrictive cardiomyopathy: Familial restrictive cardiomyopathy. Clin. Genet..

[44] Brodehl A., Gaertner-Rommel A., Klauke B., Grewe S.A., Schirmer I., Peterschröder A., Faber L., Vorgerd M., Gummert J., Anselmetti D. (2017). The novel αB-crystallin (CRYAB) mutation p.D109G causes restrictive cardiomyopathy. Hum. Mutat..

[45] Kaski J.P., Syrris P., Burch M., Tomé-Esteban M.T., Fenton M., Christiansen M., Andersen P.S., Sebire N., Ashworth M., Deanfield J.E. (2008). Idiopathic restrictive cardiomyopathy in children is caused by mutations in cardiac sarcomere protein genes. Heart.

[46] Wu W., Lu C.X., Wang Y.N., Liu F., Chen W., Liu Y.T., Han Y.C., Cao J., Zhang S.Y., Zhang X. (2015). Novel phenotype-genotype correlations of restrictive cardiomyopathy with myosin-binding protein c (mybpc3) gene mutations tested by next-generation sequencing. J. Am. Heart Assoc..

[47] Menon S.C., Michels V.V., Pellikka P.A., Ballew J.D., Karst M.L., Herron K.J., Nelson S.M., Rodeheffer R.J., Olson T.M. (2008). Cardiac troponin T mutation in familial cardiomyopathy with variable remodeling and restrictive physiology. Clin. Genet..

[48] Caleshu C., Sakhuja R., Nussbaum R.L., Schiller N.B., Ursell P.C., Eng C., De Marco T., McGlothlin D., Burchard E.G., Rame J.E. (2011). Furthering the link between the sarcomere and primary cardiomyopathies: Restrictive cardiomyopathy associated with multiple mutations in genes previously associated with hypertrophic or dilated cardiomyopathy. Am. J. Med. Genet. Part. A.

[49] Burke M.A., Cook S.A., Seidman J.G., Seidman C.E. (2016). Clinical and Mechanistic Insights into the Genetics of Cardiomyopathy. J. Am. Coll. Cardiol..

[50] Kushwaha S.S., Fallon J.T., Fuster V. (1997). Restrictive cardiomyopathy. N. Engl. J. Med..

[51] Pereira N.L., Grogan M., Dec G.W. (2018). Spectrum of Restrictive and Infiltrative Cardiomyopathies: Part 1 of a 2-Part Series. J. Am. Coll. Cardiol..

[52] Thiene G., Valente M., Angelini A. (1989). Primary restrictive cardiomyopathy: The paradox of a small heart requiring transplantation. Eur. Heart J..

[53] Geske J.B., Anavekar N.S., Nishimura R.A., Oh J.K., Gersh B.J. (2016). Differentiation of Constriction and Restriction: Complex Cardiovascular Hemodynamics. J. Am. Coll. Cardiol..

[54] Keijsers R.G.M., Grutters J.C. (2020). In Which Patients with Sarcoidosis Is FDG PET/CT Indicated?. J. Clin. Med..

[55] Ammash N.M., Seward J.B., Bailey K.R., Edwards W.D., Tajik A.J. (2000). Clinical profile and outcome of idiopathic restrictive cardiomyopathy. Circulation.

[56] Elliott P.M., Anastasakis A., Borger M.A., Borggrefe M., Cecchi F., Charron P., Hagege A.A., Lafont A., Limongelli G., Mahrholdt H. (2014). 2014 ESC Guidelines on diagnosis and management of hypertrophic cardiomyopathy: The Task Force for the Diagnosis and Management of Hypertrophic Cardiomyopathy of the European Society of Cardiology (ESC). Eur. Heart J..

[57] Limongelli G., Fratta F. (2011). S1.4 Cardiovascular involvement in Pompe disease. Acta Myol..

[58] Rauen K.A. (2013). The RASopathies. Annu. Rev. Genom. Hum. Genet..

[59] Kyle R.A., Linos A., Beard C.M., Linke R.P., Gertz M.A., O’Fallon W.M., Kurland L.T. (1992). Incidence and natural history of primary systemic amyloidosis in Olmsted County, Minnesota, 1950 through 1989. Blood.

[60] Pinney J.H., Smith C.J., Taube J.B., Lachmann H.J., Venner C.P., Gibbs S.D., Dungu J., Banypersad S.M., Wechalekar A.D., Whelan C.J. (2013). Systemic Amyloidosis in England: An epidemiological study. Br. J. Haematol..

[61] Hemminki K., Li X., Försti A., Sundquist J., Sundquist K. (2012). Incidence and survival in non-hereditary amyloidosis in Sweden. BMC Public Health.

[62] González-López E., Gallego-Delgado M., Guzzo-Merello G., de Haro-Del Moral F.J., Cobo-Marcos M., Robles C., Bornstein B., Salas C., Lara-Pezzi E., Alonso-Pulpon L. (2015). Wild-type transthyretin amyloidosis as a cause of heart failure with preserved ejection fraction. Eur. Heart J..

[63] Jacobson D.R., Alexander A.A., Tagoe C., Buxbaum J.N. (2015). Prevalence of the amyloidogenic transthyretin (TTR) V122I allele in 14 333 African-Americans. Amyloid.

[64] Muchtar E., Blauwet L.A., Gertz M.A. (2017). Restrictive cardiomyopathy: Genetics, pathogenesis, clinical manifestations, diagnosis, and therapy. Circ. Res..

[65] Cyrille N.B., Goldsmith J., Alvarez J., Maurer M.S. (2014). Prevalence and prognostic significance of low QRS voltage among the three main types of cardiac amyloidosis. Am. J. Cardiol..

[66] Mussinelli R., Salinaro F., Alogna A., Boldrini M., Raimondi A., Musca F., Palladini G., Merlini G., Perlini S. (2013). Diagnostic and prognostic value of low QRS voltages in cardiac AL amyloidosis. Ann. Noninvasive Electrocardiol..

[67] Maurer M.S., Elliott P., Comenzo R., Semigran M., Rapezzi C. (2017). Addressing common questions encountered in the diagnosis and management of cardiac amyloidosis. Circulation.

[68] Quarta C.C., Solomon S.D., Uraizee I., Kruger J., Longhi S., Ferlito M., Gagliardi C., Milandri A., Rapezzi C., Falk R.H. (2014). Left ventricular structure and function in transthyretin-related versus light-chain cardiac amyloidosis. Circulation.

[69] Nihoyannopoulos P., Dawson D. (2009). Restrictive cardiomyopathies. Eur. J. Echocardiogr..

[70] Palka P., Lange A., Donnelly J.E., Scalia G., Burstow D.J., Nihoyannopoulos P. (2002). Doppler tissue echocardiographic features of cardiac amyloidosis. J. Am. Soc. Echocardiogr..

[71] Maceira A.M., Joshi J., Prasad S.K., Moon J.C., Perugini E., Harding I., Sheppard M.N., Poole-Wilson P.A., Hawkins P.N., Pennell D.J. (2005). Cardiovascular magnetic resonance in cardiac amyloidosis. Circulation.

[72] Fontana M., Chung R., Hawkins P.N., Moon J.C. (2015). Cardiovascular magnetic resonance for amyloidosis. Heart Fail. Rev..

[73] Lewis A., Rider O. (2020). The use of cardiovascular magnetic resonance for the assessment of left ventricular hypertrophy. Cardiovasc. Diagn. Ther..

[74] Gillmore J.D., Maurer M.S., Falk R.H., Merlini G., Damy T., Dispenzieri A., Wechalekar A.D., Berk J.L., Quarta C.C., Grogan M. (2016). Nonbiopsy Diagnosis of Cardiac Transthyretin Amyloidosis. Circulation.

[75] Sethi S., Vrana J.A., Theis J.D., Leung N., Sethi A., Nasr S.H., Fervenza F.C., Cornell L.D., Fidler M.E., Dogan A. (2012). Laser microdissection and mass spectrometry-based proteomics aids the diagnosis and typing of renal amyloidosis. Kidney Int..

[76] Vrana J.A., Gamez J.D., Madden B.J., Theis J.D., Bergen H.R., Dogan A. (2009). Classification of amyloidosis by laser microdissection and mass spectrometry-based proteomic analysis in clinical biopsy specimens. Blood.

[77] Maurer M.S., Schwartz J.H., Gundapaneni B., Elliott P.M., Merlini G., Waddington-Cruz M., Kristen A.V., Grogan M., Witteles R., Damy T. (2018). Tafamidis Treatment for Patients with Transthyretin Amyloid Cardiomyopathy. N. Engl. J. Med..

[78] Koike H., Katsuno M. (2020). Transthyretin Amyloidosis: Update on the Clinical Spectrum, Pathogenesis, and Disease-Modifying Therapies. Neurol. Ther..

[79] Hagège A., Réant P., Habib G., Damy T., Barone-Rochette G., Soulat G., Donal E., Germain D.P. (2019). Fabry disease in cardiology practice: Literature review and expert point of view. Arch. Cardiovasc. Dis..

[80] Spada M., Pagliardini S., Yasuda M., Tukel T., Thiagarajan G., Sakuraba H., Ponzone A., Desnick R.J. (2006). High incidence of later-onset Fabry disease revealed by newborn screening. Am. J. Hum. Genet..

[81] Germain D.P. (2010). Fabry disease. Orphanet. J. Rare Dis..

[82] Hagège A.A., Caudron E., Damy T., Roudaut R., Millaire A., Etchecopar-Chevreuil C., Tran T.C., Jabbour F., Boucly C., Prognon P. (2011). Screening patients with hypertrophic cardiomyopathy for Fabry disease using a filter-paper test: The FOCUS study. Heart.

[83] Linhart A., Kampmann C., Zamorano J.L., Sunder-Plassmann G., Beck M., Mehta A., Elliott P.M., European FOS Investigators (2007). Cardiac manifestations of Anderson-Fabry disease: Results from the international Fabry outcome survey. Eur. Heart J..

[84] Germain D.P., Brand E., Burlina A., Cecchi F., Garman S.C., Kempf J., Laney D.A., Linhart A., Maródi L., Nicholls K. (2018). Phenotypic characteristics of the p.Asn215Ser (p.N215S) GLA mutation in male and female patients with Fabry disease: A multicenter Fabry Registry study. Mol. Genet. Genomic. Med..

[85] Nagueh S.F. (2014). Anderson-fabry disease and other lysosomal storage disorders. Circulation.

[86] Krämer J., Niemann M., Störk S., Frantz S., Beer M., Ertl G., Wanner C., Weidemann F. (2014). Relation of burden of myocardial fibrosis to malignant ventricular arrhythmias and outcomes in fabry disease. Am. J. Cardiol..

[87] Hagège A.A., Germain D.P. (2015). Adult patients with Fabry disease: What does the cardiologist need to know?. Heart.

[88] Frustaci A., Morgante E., Russo M.A., Scopelliti F., Grande C., Verardo R., Franciosa P., Chimenti C. (2015). Pathology and Function of Conduction Tissue in Fabry Disease Cardiomyopathy. Circ. Arrhythmia Electrophysiol..

[89] Acharya D., Doppalapudi H., Tallaj J.A. (2015). Arrhythmias in Fabry Cardiomyopathy. Card. Electrophysiol. Clin..

[90] Wu J.C., Ho C.Y., Skali H., Abichandani R., Wilcox W.R., Banikazemi M., Packman S., Sims K., Solomon S.D. (2010). Cardiovascular manifestations of fabry disease: Relationships between left ventricular hypertrophy, disease severity, and-galactosidase a activity. Eur. Heart J..

[91] Yeung D.F., Sirrs S., Tsang M.Y.C., Gin K., Luong C., Jue J., Nair P., Lee P.K., Tsang T.S.M. (2018). Echocardiographic Assessment of Patients with Fabry Disease. J. Am. Soc. Echocardiogr..

[92] Zamorano J., Serra V., Pérez de Isla L., Feltes G., Calli A., Barbado F.J., Torras J., Hernandez S., Herrera J., Herrero J.A. (2011). Usefulness of tissue Doppler on early detection of cardiac disease in Fabry patients and potential role of enzyme replacement therapy (ERT) for avoiding progression of disease. Eur. J. Echocardiogr..

[93] Moon J.C., Sheppard M., Reed E., Lee P., Elliott P.M., Pennell D.J. (2006). The histological basis of late gadolinium enhancement cardiovascular magnetic resonance in a patient with Anderson-Fabry disease. J. Cardiovasc. Magn. Reson..

[94] Noureldin R.A., Liu S., Nacif M.S., Judge D.P., Halushka M.K., Abraham T.P., Ho C., Bluemke D.A. (2012). The diagnosis of hypertrophic cardiomyopathy by cardiovascular magnetic resonance. J. Cardiovasc. Magn. Reson..

[95] Sado D.M., White S.K., Piechnik S.K., Banypersad S.M., Treibel T., Captur G., Fontana M., Maestrini V., Flett A.S., Robson M.D. (2013). Identification and assessment of anderson-fabry disease by cardiovascular magnetic resonance noncontrast myocardial T1 mapping. Circ. Cardiovasc. Imaging.

[96] Thompson R.B., Chow K., Khan A., Chan A., Shanks M., Paterson I., Oudit G.Y. (2013). T1 mapping with cardiovascular MRI is highly sensitive for fabry disease independent of hypertrophy and sex. Circ. Cardiovasc. Imaging.

[97] Luis S.A., Maleszewski J.J., Young P.M., Schaff H.V., Pereira N.L. (2017). Previously Unreported in Women Galactosidase Alpha Pro409Ser Variant Is Associated with Fabry Disease. Circ. Cardiovasc. Genet..

[98] Germain D.P., Weidemann F., Abiose A., Patel M.R., Cizmarik M., Cole J.A., Beitner-Johnson D., Benistan K., Cabrera G., Charrow J. (2013). Analysis of left ventricular mass in untreated men and in men treated with agalsidase-β: Data from the Fabry Registry. Genet. Med..

[99] Weidemann F., Niemann M., Breunig F., Herrmann S., Beer M., Störk S., Voelker W., Ertl G., Wanner C., Strotmann J. (2009). Long-term effects of enzyme replacement therapy on fabry cardiomyopathy. Evidence for a better outcome with early treatment. Circulation.

[100] Ortiz A., Abiose A., Bichet D.G., Cabrera G., Charrow J., Germain D.P., Hopkin R.J., Jovanovic A., Linhart A., Maruti S.S. (2016). Time to treatment benefit for adult patients with Fabry disease receiving agalsidase β: Data from the Fabry Registry. J. Med. Genet..

[101] Mehta A., Beck M., Elliott P., Giugliani R., Linhart A., Sunder-Plassmann G., Schiffmann R., Barbey F., Ries M., Clarke J.T. (2009). Enzyme replacement therapy with agalsidase alfa in patients with Fabry’s disease: An analysis of registry data. Lancet.

[102] Maron B.J., Roberts W.C., Arad M., Haas T.S., Spirito P., Wright G.B., Almquist A.K., Baffa J.M., Saul J.P., Ho C.Y. (2009). Clinical outcome and phenotypic expression in LAMP2 cardiomyopathy. JAMA.

[103] Porto A.G., Brun F., Severini G.M., Losurdo P., Fabris E., Taylor M.R.G., Mestroni L., Sinagra G. (2016). Clinical Spectrum of PRKAG2 Syndrome. Circ. Arrhythmia Electrophysiol..

[104] Murphy R.T., Mogensen J., McGarry K., Bahl A., Evans A., Osman E., Syrris P., Gorman G., Farrell M., Holton J.L. (2005). Adenosine monophosphate-activated protein kinase disease mimicks hypertrophic cardiomyopathy and Wolff-Parkinson-White syndrome: Natural history. J. Am. Coll. Cardiol..

[105] Bayrak F., Komurcu-Bayrak E., Mutlu B., Kahveci G., Basaran Y., Erginel-Unaltuna N. (2006). Ventricular pre-excitation and cardiac hypertrophy mimicking hypertrophic cardiomyopathy in a Turkish family with a novel PRKAG2 mutation. Eur. J. Heart Fail..

[106] Gollob M.H., Seger J.J., Gollob T.N., Tapscott T., Gonzales O., Bachinski L., Roberts R. (2001). Novel PRKAG2 mutation responsible for the genetic syndrome of ventricular preexcitation and conduction system disease with childhood onset and absence of cardiac hypertrophy. Circulation.

[107] Blair E., Redwood C., Ashrafian H., Oliveira M., Broxholme J., Kerr B., Salmon A., Ostman-Smith I., Watkins H. (2001). Mutation in the γ2 subunit of AMP-activated protein kinase cause familial hypertrophic cardiomyopathy: Evidence for the central role of energy compromise in disease pathogenesis. Hum. Mol. Genet..

[108] Pöyhönen P., Hiippala A., Ollila L., Kaasalainen T., Hänninen H., Heliö T., Tallila J., Vasilescu C., Kivistö S., Ojala T. (2015). Cardiovascular magnetic resonance findings in patients with PRKAG2 gene mutations. J. Cardiovasc. Magn. Reson..

[109] Forsha D., Li J.S., Smith P.B., Van Der Ploeg A.T., Kishnani P., Pasquali S.K. (2011). Cardiovascular abnormalities in late-onset Pompe disease and response to enzyme replacement therapy. Genet. Med..

[110] Olson L.J., Reeder G.S., Noller K.L., Edwards W.D., Howell R.R., Michels V.V. (1984). Cardiac involvement in glycogen storage disease III: Morphologic and biochemical characterization with endomyocardial biopsy. Am. J. Cardiol..

